# Local administration of granulocyte macrophage colony-stimulating factor induces local accumulation of dendritic cells and antigen-specific CD8+ T cells and enhances the efficacy of therapeutic vaccine in cervicovaginal tumor

**DOI:** 10.1186/2051-1426-3-S2-P365

**Published:** 2015-11-04

**Authors:** Sung-Jong Lee, TC Wu, Chien-Fu Hung

**Affiliations:** 1 St. Vincent's Hospital, The Catholic University of Korea, Bethesda, MD, USA; 2 Johns Hopkins Medical Institution, Baltimore, MD, USA

## 

Immunotherapy has emerged as a promising treatment strategy for the control of HPV-associated malignancies. Various therapeutic HPV vaccines have elicited potent antigen-specific CD8+ T cell mediated antitumor immune responses in preclinical models and are currently being tested in several clinical trials. Recent evidence has reported the importance of local immune activation, and higher number of immune cells in the site of lesion correlates with positive prognosis. Granulocyte macrophage colony-stimulating factor (GMCSF) has been reported to possess the ability to induce migration of antigen presentation cells and CD8+ T cells. Therefore, in the current study, we employed a combination of systemic therapeutic HPV DNA vaccination with local GMCSF application in the TC-1 tumor. We show that intramuscular vaccination with CRT/E7 DNA followed by GMCSF intravaginal administration effectively controls TC-1 tumor in mice. Furthermore, we observe an increase in the accumulation of E7-specific CD8+ T cells and dendritic cells in the vaginal tumor following the combination treatment. In addition, we show that GMCSF induces activation and maturation in dendritic cells and promote antigen cross-presentation. Our results support the clinical translation of the combination treatment of systemic therapeutic vaccination followed by local GMCSF administration as an effective strategy for tumor treatment.

**Figure 1:**
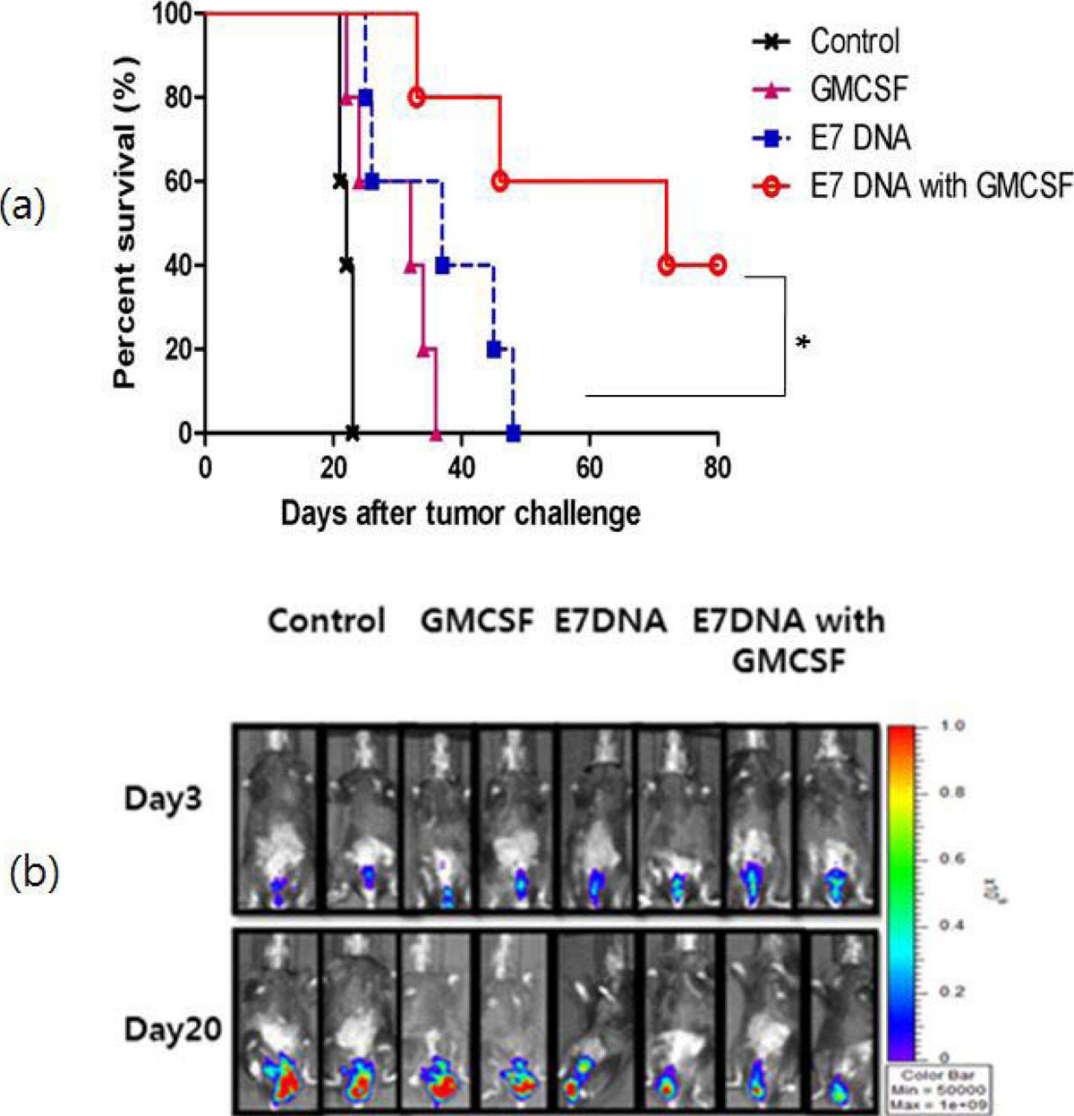
(a) Survival curve analysis of four groups (b) Bioluminescence of tumor growth.

**Figure 2:**
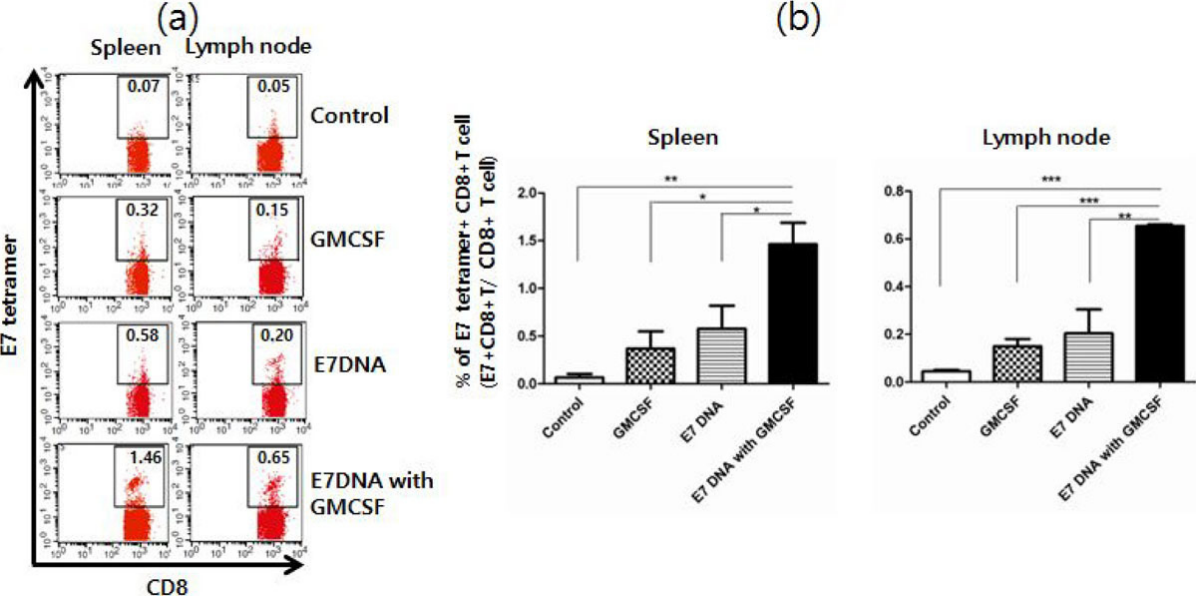
(a) Percentage of E7-specific CD8+ T cells in spleen and lymph node in tumor bearing mice (b) Bar graph explaining E7-specific CD8+ T cells in spleen and lymph node in tumor bearing mice

**Figure 3:**
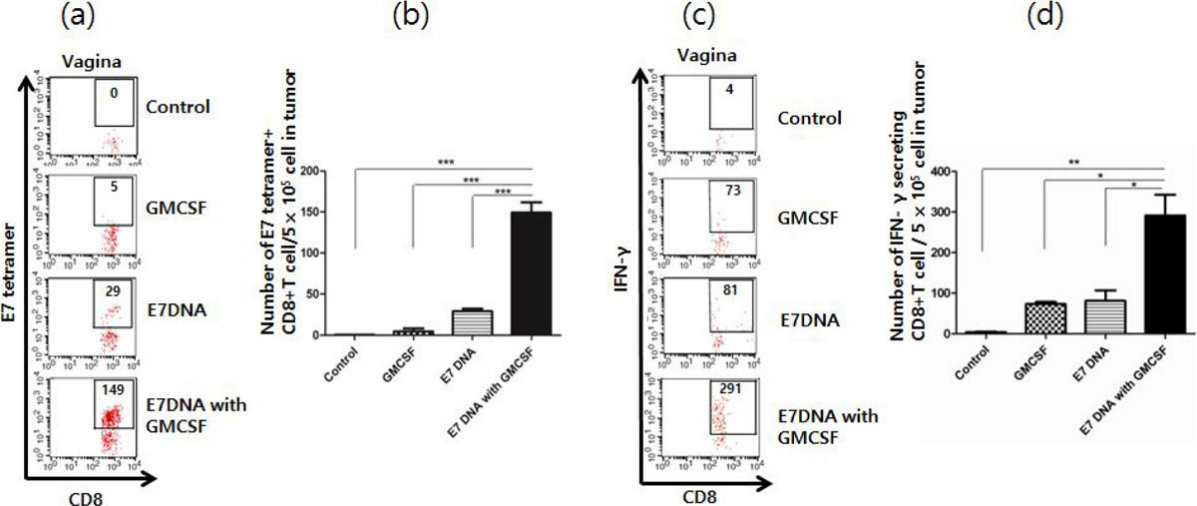
(a) The number of E7-specific CD8+ T cells in cervicovaginal tumor of four groups (b) Bar graph depicting the number of E7-specific CD8+ T cells (c) The number of IFN-y positive CD8+ T cells per 5 × 10^5 tumor cells (d) Bar graph depicting the number of IFN-y positive CD8+ T cells per 5 × 10^5 tumor cells

**Figure 4:**
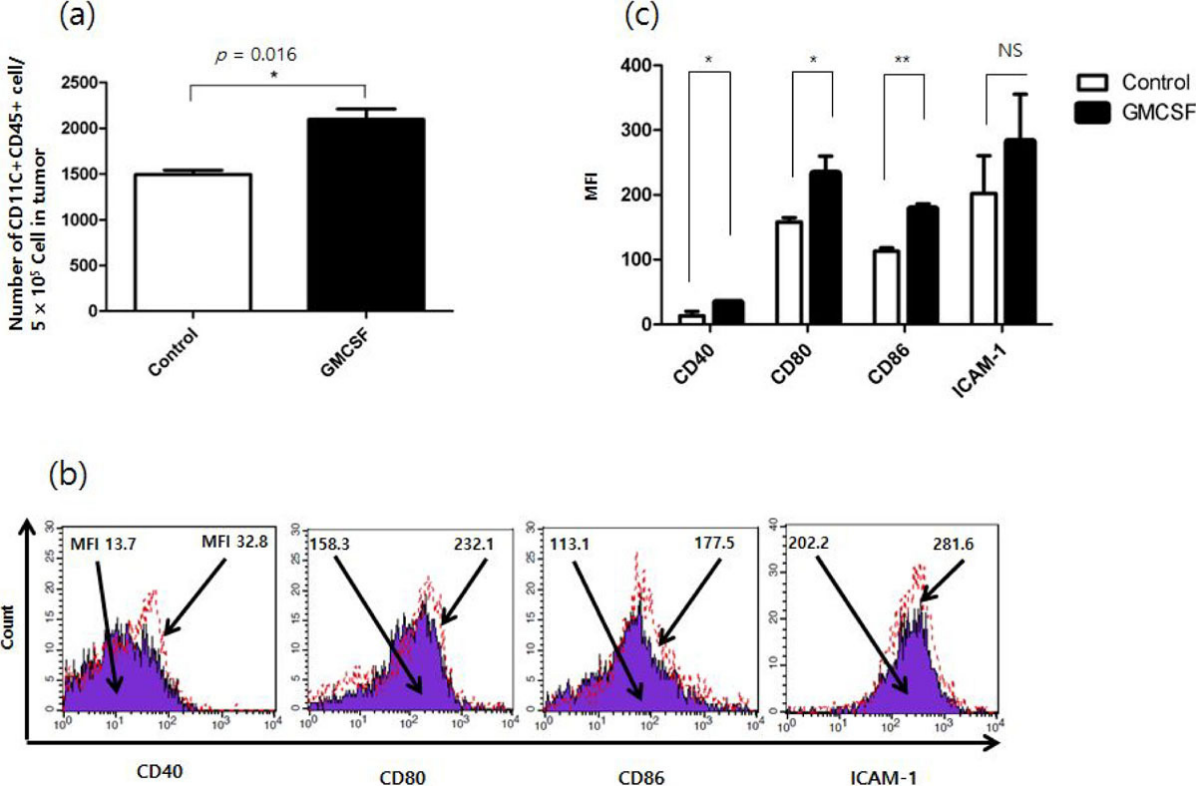
(a) The number of dendritic cells (CD11C+CD45+) per 5 × 10^5 tumor cells (b,c) Maturation markers of dendritic cells (CD11C+CD45+) in vaginal tumor.

**Figure 5:**
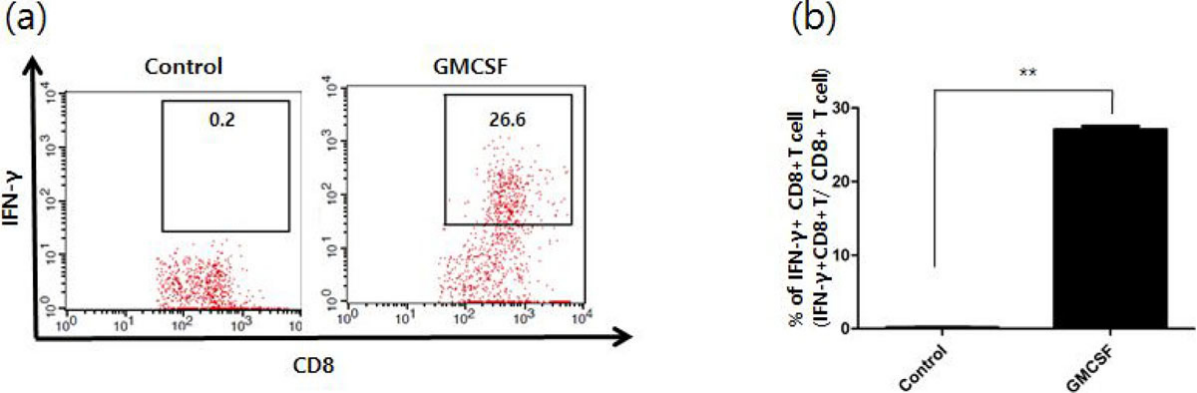
1 × 10^5 E7-Specific CD8 T cell were cultured together with isolated dendritic cells in 1:1 ratio for 12 hours and stained with FITC-conjugated IFN-y and APC-conjugated CD8 followed by flow cytometry analysis (a) Representative data of IFN-y staining followed by flow cytometry analysis showing the percentage of IFN-y positive CD8+ T cells (b) Bar graph depicting the percentage of IFN-y positive CD8+ T cells.

